# Effect of hydroalcoholic extract of *Myracrodruon urundeuva* All. and *Qualea grandiflora* Mart. leaves on the viability and activity of microcosm biofilm and on enamel demineralization

**DOI:** 10.1590/1678-7757-2018-0514

**Published:** 2019-05-30

**Authors:** Juliana Gonçalves Pires, Aline Silva Braga, Flaviana Bombarda de Andrade, Luiz Leonardo Saldanha, Anne Lígia Dokkedal, Rodrigo Cardoso de Oliveira, Ana Carolina Magalhães

**Affiliations:** 1 Universidade de São Paulo, Faculdade de Odontologia de Bauru, Departamento de Ciências Biológicas, Bauru, São Paulo, Brasil.; 2 Universidade de São Paulo, Faculdade de Odontologia de Bauru, Departamento de Dentística, Endodontia e Materiais Odontológicos, Bauru, São Paulo, Brasil.; 3 Universidade Estadual Paulista (UNESP), Faculdade de Ciências, Departamento de Ciências Biológicas, Bauru, São Paulo, Brasil.

**Keywords:** Antimicrobial agents, Dental caries, Biofilms, Plant extracts, Phytotherapy

## Abstract

**Objectives:**

The aim of this study was to assess the effect of *Myracrodruon urundeuva* All. and *Qualea grandiflora* Mart. leaves hydroalcoholic extracts on viability and metabolism of a microcosm biofilm and on enamel demineralization prevention.

**Methodology:**

Microcosm biofilm was produced on bovine enamel using inoculum from pooled human saliva mixed with McBain saliva, under 0.2% sucrose exposure, for 14 days. The biofilm was daily-treated with the extracts for 1 min. At the end, it was analyzed with respect to viability by fluorescence, CFU counting and extracellular polysaccharides (phenol-sulphuric acid colorimetric assay) and lactic acid (enzymatic assay) production. The demineralization was measured by TMR. The data were compared using ANOVA or Kruskal-Wallis (p<0.05).

**Results:**

*M. urundeuva* All. at 100, 10 and 0.1 μg/mL and *Q. grandiflora* Mart. at 100 and 0.1 μg/mL reduced biofilm viability similarly to positive control (chlorhexidine) and significantly more than the negative-vehicle control (35% ethanol). *M. urundeuva* at 1000, 100 and 0.1 μg/mL were able to reduce both lactobacilli and *mutans streptococci* CFU counting, while *Q. grandiflora* (1000 and 1.0 μg/mL) significantly reduced *mutans streptococci* CFU counting. On the other hand, the natural extracts were unable to significantly reduce extracellular polysaccharides and lactic acid productions neither the development of enamel carious lesions.

**Conclusions:**

The extracts showed antimicrobial properties on microcosm biofilm, however, they had no effect on biofilm metabolism and caries protection.

## Introduction

Dental caries involves dental biofilm rich in acidogenic and aciduric bacteria such as *Streptococcus mutans, Streptococcus sobrinus*, *Lactobacillus* sp., *Veillonella*, *Actinomyces*, bifidobacteria and fungi,^1^ which are metabolically active under frequent sugar exposure, producing acids that induce tooth demineralization.^2^ Mechanical disorganization of dental biofilm by brushing and rationing sugar consumption are key strategies to prevent the disease. In addition, conventional antimicrobial oral mouthrinses can be recommended for patients at high-risk level.^3^ However, their antimicrobial properties may not reflect into an anti-caries effect and, additionally, may induce some side-effects such as taste alteration, tooth staining and mucosa desquamation.^4^ ^,^ ^5^ Therefore, scientists are directing attention to folk medicine in order to find alternative antimicrobial agents against oral diseases as dental caries.^6^

Brazil is the country harboring the highest plant diversity, allocated mainly in Cerrado and the Atlantic Forest.^7^ *Myracrodruon urundeuva* All. (Anacardiaceae) and *Qualea grandiflora* Mart. (Vochysiaceae) are examples of plants from Brazilian Cerrado.

*M. urundeuva* has antimicrobial action,^8^ ^,^ ^9^ including action against *mutans streptococci*,^10^ as well as analgesic, hepatoprotective, antidiarrheal, colonic anastomotic wound healing and anti-ulcerogenic effects.^11^ *Q. grandiflora* exhibits anti-ulcerogenic action in the ethanolic extract of its bark.^12^ Besides, this extract has an antioxidant effect,^13^ analgesic and anticonvulsive potential^14^ and antibacterial action.^15^

Regarding dental caries, a previous study tested the effect of aqueous extracts of *M. urundeuva* on *mutans streptococci* counts and on dental enamel micro-hardness of rats submitted to cariogenic challenges. The extract promoted significant reduction of *mutans streptococci* counts as well as enamel demineralization.^16^

Recently, our research group showed that both hydroalcoholic extracts of *M. urundeuva* and *Q. grandiflora* leaves (isolated or combined) had antimicrobial action; however, they did not prevent enamel caries formation under the *mutans streptococci* biofilm model.^17^ Therefore, there is no consensus about the anti-caries action of the extracts. Furthermore, there is no information about their mechanism of action under more complex biofilm models (such as multispecies or microcosm biofilm).

Considering the need for alternatives to prevent dental caries in specific populations that are under unfavorable socioeconomic conditions,^18^ the aim of our study was to evaluate the effect of hydroalcoholic extracts of *M. urundeuva* and *Q. grandiflora* leaves on the viability and metabolism of a microcosm biofilm and on the prevention of enamel demineralization.

## Methodology

### Saliva collection

This study was approved by the local Ethics Committee (CEEA 43948115.2.0000.5417). After consent, the saliva pool collected from 2 healthy donors who followed the inclusion criteria previously described by Souza, et al.^19^ (2018) was mixed with glycerol and frozen.

### Plant material preparation

Leaf samples of *M. urundeuva* and *Q. grandiflora* were collected in October 2013 at the Jardim Botânico Municipal de Bauru (Bauru, Brazil), (22°20′41.4″S - 49°01′45.1″W). Exsiccates were deposited in the Herbarium of UNESP under code numbers HRCB59831 and UNBA6034. The collections have authorization issued by SISBIO under code number 39825-1. The leaves' extracts were prepared as described by Machado, et al.^20^ (2016).

### Tooth sample preparation and treatment groups

Three hundred and six enamel samples (4 mm x 4 mm) were prepared from bovine teeth, following the study by Braga, Pires and Magalhães^5^ (2018). Sample size was calculated based on a previous study.^17^ The samples were sterilized using ethylene oxide [gas exposure time (30% ETO/70%CO_2_) for 4 h under a pressure of 0.5±0.1 kgF/cm^2^].

The enamel samples were randomly divided into treatment groups by using their average roughness-Ra means (Ra: 0.153±0.037 μm) as criteria, presented as follows: PerioGard^®^ with alcohol (0.12% chlorhexidine digluconate, Colgate; São Bernardo do Campo, São Paulo, Brazil) – Positive control (pH 5.0); 35% ethanol –Negative/Vehicle control (pH 5.7); hydroalcoholic extracts from the leaves of *M. urundeuva* at 0.1 (pH 5.7); 1.0 (pH 5.8); 10 (pH 5.2); 100 (pH 5.2) and 1000 μg/mL (pH 4.8) and *Q. grandiflora* at 0.1 (pH 5.3); 1.0 (pH 5.4); 10 (pH 5.1); 100 (pH 4.9) and 1000 μg/mL (pH 4.5). All extract solutions contained 35% alcohol as solvent.

### Microcosm biofilm formation and treatments

The human saliva was defrosted and mixed with McBain saliva^21^ in a proportion of 1:50. The microcosm biofilm was produced as described in previous studies.^5^ ^,^ ^19^ The samples were placed in a 24-well plate and the solution containing human saliva and McBain saliva was added to each well (v=1.5 mL/well), which was incubated at 5% CO_2_ and 37°C for the first 8 h. Thereafter, the samples were washed with PBS and exposed to fresh McBain saliva with 0.2% sucrose and incubated until completing the 1^st^ day, at the same conditions.

From the 2^nd^ to the 14^th^ day, the samples were treated once a day with natural agents or controls for 1 min (1 mL/well) at room temperature. Afterwards, the samples were washed using PBS, and fresh McBain saliva containing 0.2% sucrose was added. The microplates were then incubated at 37°C and 5% CO_2_.^22^

### Biofilm viability analysis

The biofilm was stained using the Kit Live & Dead^®^ cells viability assay (Thermo Fisher Scientific; Waltham, Massachusetts, USA).^17^ The biofilm was examined using confocal laser scanning microscope-CLSM (Leica TCS SPE; Mannheim, Baden-Württemberg, Germany) and Leica Application Suite-Advanced Fluorescence software (LAS AF; Mannheim, Baden-Württemberg, Germany). Three images (275 μm^2^) were captured and analyzed using the BioImage L 2.0 application software to quantify the live and dead bacteria (%).

### Microorganism viability analysis

For colony-forming unit CFU counting, 100 μl of the bacterial suspension was diluted to 10^-4^ and spread on petri dishes (25 μl/dish) containing two different types of agar: A) SB-20M^23^ for determination of *mutans streptococci* (*S. mutans* and *S. sobrinus*); and B) Rogosa (Kasvi; Curitiba, Paraná, Brazil) supplemented with 0.13% glacial acetic acid to assess the number of lactobacilli.^24^ The plates were then incubated at 5% CO_2_ and 37°C. After 48 h, the CFU numbers were counted and transformed in log_10_ CFU/mL.

### Metabolism analysis

#### a) Lactic acid production

For this assay, only the highest and lowest concentrations of each extract were tested. Lactate concentrations were evidenced by means of the enzymatic method (lactic dehydrogenase method, Boehringer; Mannheim, Baden-Württemberg, Germany) according to the manufacturer's instruction.^25^ Absorbance was measured at 340 nm using a microplate reader (Fluorstar Optima- BMG Labtech; Ortenberg, Baden-Württemberg, Germany). The values were expressed as mmol lactate/L.^5^

#### b) Extracellular polysaccharides – EPS quantification

The insoluble and soluble EPS were quantified as previously performed.^5^ Total carbohydrates were measured using the phenol-sulphuric acid colorimetric assay under absorbance of 490 nm using a microplate reader (Fluorstar Optima- BMG Labtech; Ortenberg, Baden-Württemberg, Germany).^26^ The values for both EPS were expressed as μg EPS/mg (biofilm).^5^

### Transverse microradiography (TMR)

Enamel slices with 80-100 μm of thickness were fixed in a sample-holder together with an aluminum calibration step wedge with 14 steps. Microradiographs were taken using an x-ray generator (Softex; Tokyo, Honshu, Japan) on the glass plates.^17^ The glass plates were developed and analyzed using a transmitted light microscope fitted with a 20x objective (Zeiss; Oberkochen, Baden-Württemberg, Germany), a CCD camera (Canon; Tokyo, Honshu, Japan), and a computer containing software from the Inspektor Research System bv (Amsterdam, North Holland, The Netherlands). The cavitation depth (CD, μm) was calculated as previously described.^17^ ^,^ ^19^

### Statistical analysis

All experiments were performed in biological triplicate (except the lactate assay, in duplicate) with three data points for each replicate. Data were statistically analyzed using the application software Graph Pad Instat for Windows (GraphPad Software; San Diego, California, USA). Normal distribution and homogeneity were checked using Kolmogorov & Smirnov and Bartlett's tests, respectively. The % live and dead microorganisms were compared using ANOVA and Tukey-Kramer test. For the remaining analyses, Kruskal-Wallis followed by Dunn test was applied. The level of significance was set at 5%.

## Results

### Bacterial viability

**Figure 1:**
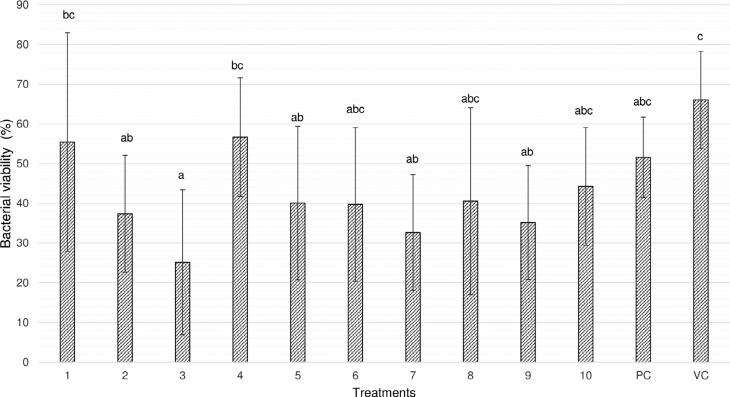
Mean±SD of the percentage (%) of live microorganisms (viability assay using CLSM) from microcosm biofilm treated with hydroalcoholic extracts of *M. urundeuva* All. and *Q. grandiflora* Mart. leaves 1- 5: *M. urundeuva* from 1000 to 0.1 μg/mL respectively; 6-10: *Q. grandiflora* from 1000 to 0.1 μg/mL respectively; PC: Positive control (Chlorhexidine, PerioGard^®^); VC: Vehicle (negative) control. Different letters show significant differences between treatments (ANOVA/Tukey-Kramer, p<0.0001)

**Figure 2:**
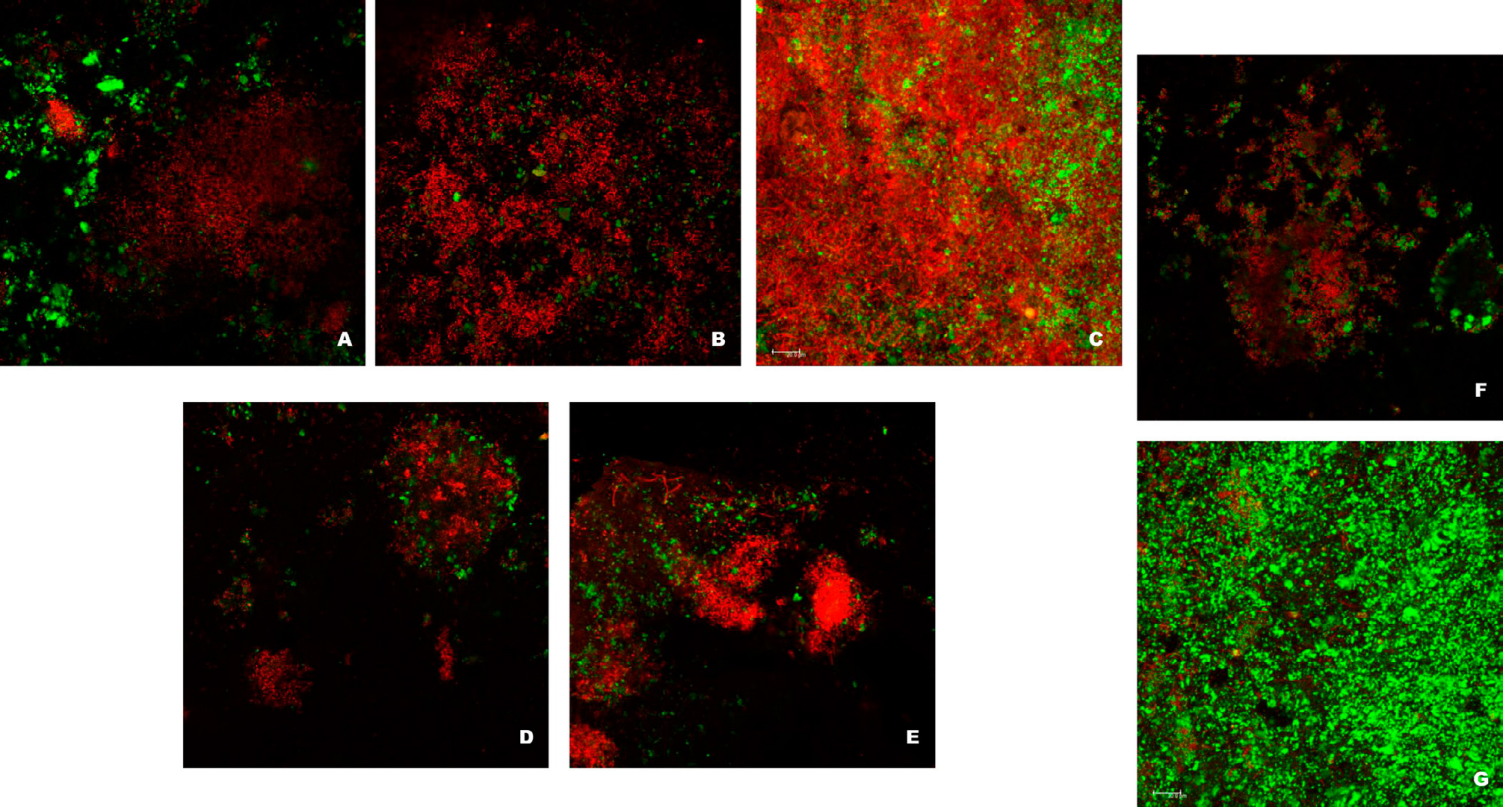
Representative image of the CLSM analysis from the groups: A-C) *M. urundeuva* at 100, 10 and 0.1 μg/mL, respectively; D-E) *Q. grandiflora* at 100 and 1 μg/mL, respectively; F) Positive control (chlorhexidine, PerioGard^®^); G) Vehicle (negative) control

Hydroalcoholic extracts of *M. urundeuva* at 100 μg/mL (62.14%), 10 μg/mL (74.59%) and 0.1 μg/mL (59.81%) and *Q. grandiflora* at 100 μg/mL (67.19%) and 1 μg/mL (64.50%) presented mean percentage of dead cells similar to the positive control (chlorhexidine, 48.21%), and significantly higher than the negative control group (35% ethanol, 33.79%). The other experimental groups did not differ between themselves and positive and negative controls (*p*>0.05, Figures 1 and 2). Figure 1 shows the percentage of viable microorganisms from each treatment's group. Figure 2 shows CLSM pictures of a representative biofilm sample from the most effective antimicrobial concentrations of the tested extracts.

### Microorganism viability

Table 1 shows the CFU counting results. With respect to lactobacilli, only *M. urundeuva* at 1000, 100 and 0.1 μg/mL were able to reduce the CFU counting similarly to positive control and significantly more compared to negative control. *M. urundeuva* at similar concentrations had the same effect on *mutans streptococci*. Despite having no effect on lactobacilli, *Q. grandiflora* at 1000 and 0.1 μg/mL significantly reduced the number of *mutans streptococci* compared to negative control. Chlorhexidine significantly reduced CFU counting for both microorganisms compared to negative control.

**Table 1:** Median (interquartile interval) of CFU counting (log_10_ CFU/mL) for lactobacilli and *mutans streptococci*

Treatments	lactobacilli	*mutans streptococci*
35% Alcohol (vehicle/negative control)	7.34(0.62)^c^	7.60(0.61)^c^
Chlorhexidine (positive control)	6.72(1.09)^ab^	6.64(1.44)^ab^
*M. urundeuva* 1000 μg/ml	6.81(0.54)^a^	6.75(0.53)^ab^
*M. urundeuva* 100 μg/ml	6.78(0.61)^ab^	6.79(0.90)^ab^
*M. urundeuva* 10 μg/ml	7.02(0.60)^abc^	6.79(0.59)^abc^
*M. urundeuva* 1.0 μg/ml	7.51(0.40)^bc^	7.45(0.75)^bc^
*M. urundeuva* 0.1 μg/ml	6.78(0.89)^a^	6.25(0.54)^a^
*Q. grandiflora* 1000 μg/ml	7.19(0.15)^abc^	6.86(0.97)^ab^
*Q. grandiflora* 100 μg/ml	7.43(0.56)^bc^	7.57(1.06)^c^
*Q. grandiflora* 10 μg/ml	6.98(0.52)^abc^	7.02(1.15)^abc^
*Q. grandiflora* 1.0 μg/ml	7.15(0.64)^abc^	7.16(0.78)^bc^
*Q. grandiflora* 0.1 μg/ml	6.88(0.69)^abc^	6.81(0.51)^ab^

* Different superscript letters at the same column show significant differences between treatments (Kruskal-Wallis/Dunn: p<0.0001 for both)

### Metabolism analysis

#### a) Lactic acid production

**Figure 3:**
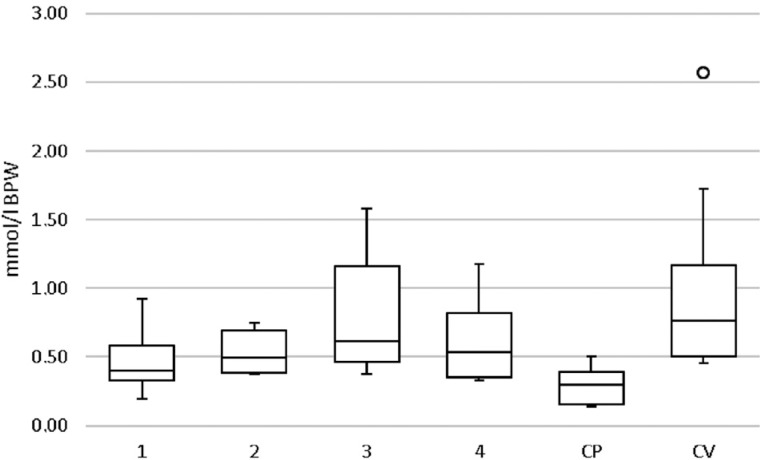
Boxplot of the lactic acid production (mmol/L BPW) using lactic dehydrogenase method 1-2: *M. urundeuva* at 1000 and 0.1 μg/mL, respectively; 3-4: *Q. grandiflora* at 1000 and 0.1 μg/mL, respectively; PC: Positive control (chlorhexidine, PerioGard^®^); VC: Vehicle (negative) control; °: Outliers. (Kruskal-Wallis/Dunn, p=0.0121)

None of the extracts was able to significantly reduce lactic acid production compared to negative control; however, chlorhexidine significantly differed from negative control (Figure 3).

#### b) EPS quantification

Table 2 shows that none of the extracts was able to significantly reduce EPS production compared to the negative control, while chlorhexidine significantly reduced soluble EPS compared to negative control.

**Table 2:** Median (interquartile interval) of the soluble and insoluble EPS (μg/mg biofilm)

Treatments	Soluble EPS (μg/mg)	Insoluble EPS (μg/mg)
35% Alcohol (vehicle/negative control)	0.22(0.06)^b^	0.32(0.17)^b^
Chlorhexidine (positive control)	0.07(0.05)^a^	0.35(0.19)^ab^
*M. urundeuva* 1000 μg/ml	0.15(0.10)^ab^	0.45(0.28)^ab^
*M. urundeuva* 100 μg/ml	0.22(0.16)^ab^	0.79(0.65)^ab^
*M. urundeuva* 10 μg/ml	0.12(0.04)^ab^	0.38(0.24)^ab^
*M. urundeuva* 1.0 μg/ml	0.14(0.07)^ab^	0.59(0.28)^ab^
*M. urundeuva*a 0.1 μg/ml	0.25(0.15)^b^	0.50(0.23)^ab^
*Q. grandiflora* 1000 μg/ml	0.28(0.13)^b^	0.87(0.44)^a^
*Q. grandiflora* 100 μg/ml	0.16(0.06)^ab^	0.52(0.19)^ab^
*Q. grandiflora* 10 μg/ml	0.17(0.07)^ab^	0.44(0.33)^ab^
*Q. grandiflora* 1.0 μg/ml	0.14(0.08)^ab^	0.52(0.21)^ab^
*Q. grandiflora* 0.1 μg/ml	0.17(0.09)^ab^	0.74(0.42)^ab^

* Different superscript letters at the same column show significant differences between treatments (Kruskal-Wallis/Dunn, p<0.0001 and p=0.0082, respectively)

### TMR

**Figure 4:**
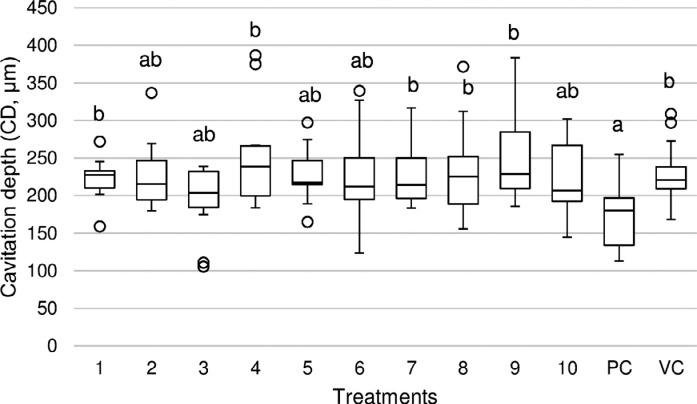
Boxplot of the cavitation depth (CD, μm) of the artificial enamel lesions created under microcosm biofilm model after applying the tested treatments 1- 5: *M. urundeuva* from 1000 to 0.1 μg/mL respectively; 6-10: *Q. grandiflora* from 1000 to 0.1 μg/mL, respectively; PC: Positive control (chlorhexidine, PerioGard^®^); VC: Vehicle (negative) control; °: Outliers. Different letters show significant differences among the treatments (Kruskal-Wallis/Dunn, p=0.0012)

**Figure 5:**
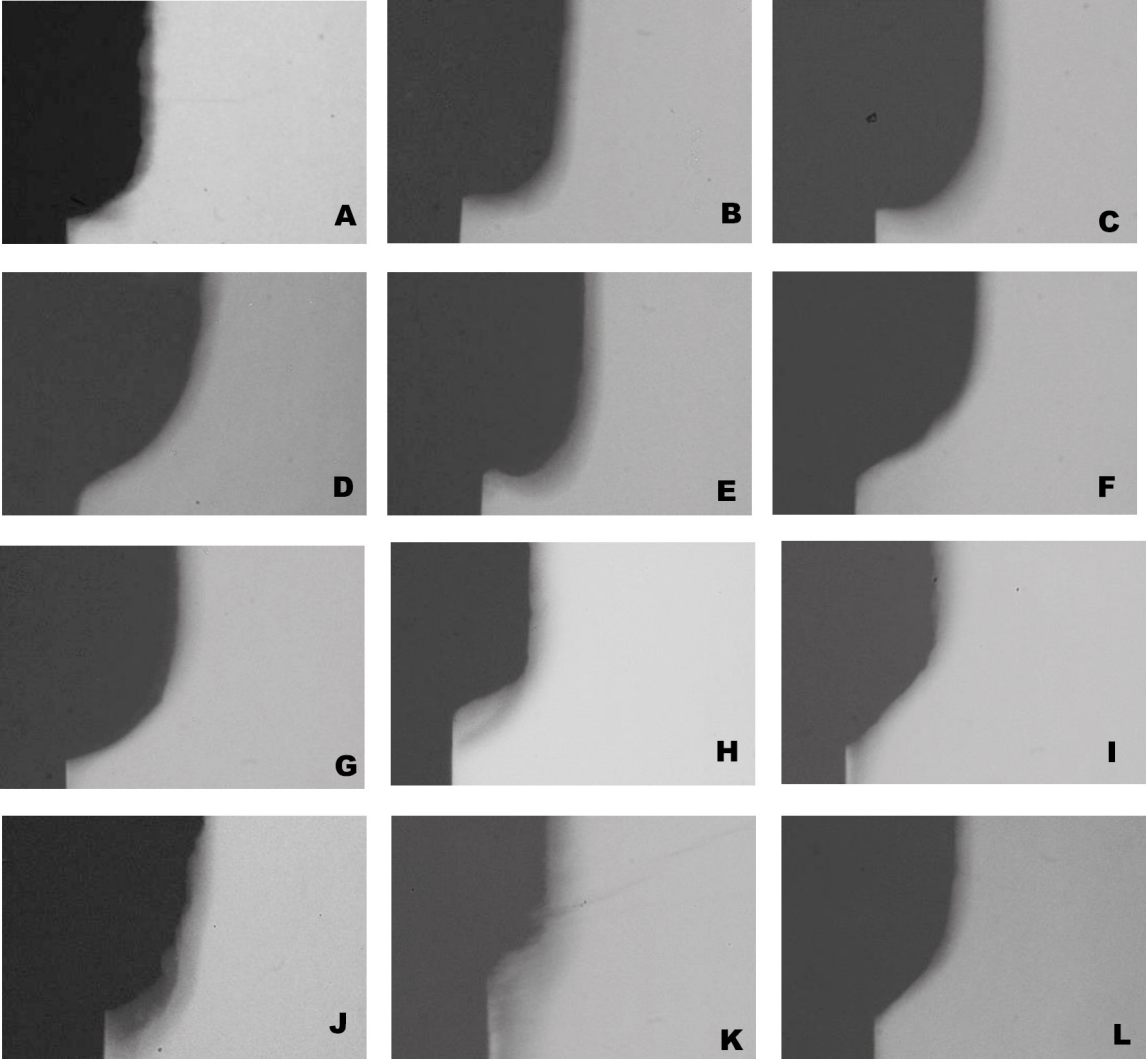
Representative TMR pictures (20x) of artificial enamel lesions created using microcosm biofilm after applying the tested treatments A-E) *M. urundeuva* from 1000 to 0.1 μg/mL, respectively; F-J) *Q. grandiflora* from 1000 to 0.1 μg/mL, respectively; K) Positive control (chlorhexidine, PerioGard^®^); L) Vehicle (negative) control

Enamel cavitation was seen in all groups with different cavitation depth values as shown in Figures 4 and 5. None of the extracts was able to reduce cavitation depth, while chlorhexidine significantly reduced cavitation depth compared to the negative control (Figure 4). Figure 5 shows TMR pictures of a representative enamel sample from each treatment's group.

## Discussion

The use of plant extracts is a common practice in worldwide medicine, since phytotherapy is considered low cost and widely accessible.^7^ Brazil is one of the countries with the greatest biodiversity,^7^ ^,^ ^27^ a fact which in turn has stimulated the use of different types of plants for prevention and treatment of oral diseases based on their antimicrobial properties.^7^ ^–^ ^10^ ^,^ ^15^ ^,^ ^17^

The experimental model for studying the effect of plants on dental caries prevention must include assays that are capable of showing both 1) antimicrobial properties and mechanism of action (such as viability, EPS and lactic acid assays) and 2) the anti-caries effect (TMR), since one is not synonym to the other. Some known antimicrobial agents have no anti-caries potential,^5^ contraindicating their use for preventing the disease. Accordingly, we have chosen a microcosm biofilm model produced on enamel that is able to simulate the heterogeneity and variability of an *in vivo* biofilm, allowing for the analysis of both biofilm and tooth.^28^ The microcosm biofilm model is able to produce reproducible biofilms that are representative of oral microbia (60% of the species from the original inoculum are preserved),^29^ however, it does not allow for checking the effect of antimicrobial agents on specific microorganisms. Furthermore, the continuous sugar exposure during 14 days produced a very aggressive biofilm inducing enamel cavitation, as can be seen in the TMR pictures (Figure 5).

*M. urundeuva* and *Q. grandiflora* were chosen as they are easily found in Brazilian Cerrado. The ethanolic extracts of *M. urundeuva* leaves and bark have as active components gallic acid, methyl gallate, ethyl gallate, chlorogenic and protocatechuic acid, saponins, flavonoids, tannins and polyphenols.^30^ The ethanolic extracts of *Q. grandiflora* leaves present gallic and ellagic acids derivatives, galotannins, ellagitannins, triterpenes, flavonoids, benzoquinones and anthraquinones.^31^ A previous study showed that the main components of the *M. urundeuva* extract are flavonoids and tannins,^20^ which are related to its anti-inflammatory and antimicrobial properties.^32^ ^,^ ^33^

Generally, our study showed that *M. urundeuva* has superior antimicrobial effect compared to *Q. grandiflora* in agreement with a previous study,^17^ which might be due to its high content of tannins and polyphenols. Alves, et al.^10^ (2009) found Minimum Inhibitory Concentration and Minimum Inhibitory Adhesion Concentration values of 0.125 mg/mL and 0.0625 mg/mL against *mutans streptococci*, respectively. Their MIC value is in agreement with our biofilm viability results, since we have seen antimicrobial effect with 0.1 mg/mL *M. urundeuva.* On the other hand, Pires, et al.^17^ (2018) showed antimicrobial effects at higher concentrations (*M. urundeuva* ≥0.625 mg/mL and *Q. grandiflora* at 5 mg/mL), which may be due to the biofilm model (3-days *mutans streptococci* biofilm) applied in their study.

With respect to *Q. grandiflora*, most studies have tested its effect on non-cariogenic bacteria such as *Staphylococcus aureus*, *Escherichia coli*, *Bacillus cereus*, *Pseudomonas aeruginosa*, *Streptococcus pyogenes* and *Helicobacter pylori*.^15^ ^,^ ^34^ The first study dealing with the anti-caries effect of *Q. grandiflora* was recently done by Pires, et al.^17^ (2018). Differently from our study, Pires, et al.^17^ (2018) only found antimicrobial effect of *Q. grandiflora* against *mutans streptococci* at 5 mg/mL, which might be due to differences in the biofilm model between both studies (monospecies biofilm vs. microcosm biofilm) as discussed above.

Despite the extracts being able to reduce bacteria viability as well as the number of lactobacilli and *mutans streptococci*, they did not interfere in biofilm metabolism, and, therefore, they were unable to reduce caries lesions development, which corroborates with a previous study.^17^ Despite the treatments having reduced the number of viable bacteria, the microorganisms were still able to produce acid and EPS, which in turn induced enamel demineralization similar to the negative control. Our work provided support for the statement that not all antimicrobial agents have anti-caries potential.^5^ Furthermore, we could not find a dose-response with respect to viability and, therefore, the antimicrobial effect of natural agents might have their biological relevance questioned.

On the other hand, it is important to emphasize that other bacteria not analyzed in the present study could have contributed to enamel caries development (*Scardovia wiggsiae*, *Bifidobacterium* spp. and *Actinomyces* spp.),^35^ which shall be further confirmed under this model in future studies.

In disagreement, a previous study has shown that an aqueous solution of *M. urundeuva* protected against enamel surface cross-sectional hardness loss in Wistars rats inoculated with *mutans streptococci*, after 7 weeks of cariogenic challenges.^16^ The different result found in the cited study might be due to the greater concentration of the extract (7.5 mg/mL) as well as the type of extract (aqueous) applied by a previous study and to the low velocity of caries development *in vivo*. It is also important to consider that a hardness assay is unable to show if the cariogenic challenges induced tooth cavitation,^36^ which is considered a limitation of the method.

Further studies shall give attention to test the antimicrobial effect of *M. urundeuva* extracts, varying concentrations, solvents and vehicles, under microcosm biofilm or *in situ* model, to confirm the possible absence of anti-caries effect. Other important point to be considered in future studies is the analysis of the cytotoxic and biological effect of the plants extracts as well.^20^ ^,^ ^33^

## Conclusions

The extracts showed antimicrobial effects (especially *M. urundeuva*) on the microcosm biofilm; however, no effect was observed on the biofilm metabolism and neither anti-caries effect under this biofilm model.
